# Morphological Changes and Expressions of *AOX1A*, *CYP81D8*, and Putative *PFP* Genes in a Large Set of Commercial Maize Hybrids Under Extreme Waterlogging

**DOI:** 10.3389/fpls.2019.00062

**Published:** 2019-02-04

**Authors:** Anna Panozzo, Cristian Dal Cortivo, Manuel Ferrari, Bianca Vicelli, Serena Varotto, Teofilo Vamerali

**Affiliations:** Department of Agronomy, Food, Natural Resources, Animals and the Environment, University of Padua, Padua, Italy

**Keywords:** hypoxia, gene expression, maize hybrids, root length, shoot biomass, SPAD

## Abstract

Waterlogging is a severe abiotic stressor causing significant growth impairment and yield losses in many crops. Maize is highly sensitive to the excess of water, and against the background of climate change there is an urgent need for deeper insights into the mechanisms of crop adaptation to waterlogging. In the present study, changes in maize morphology at the 4–5 leaf stage and the expression of three candidate genes for flooding tolerance in plants subjected to six continuous days of waterlogging were recorded in 19 commercial hybrids and in the inbred line B73, with the aim of investigating the current variability in cultivated hybrids and identifying useful morphological and molecular markers for screening tolerant genotypes. Here it was demonstrated that root parameters (length, area, biomass) were more impaired by waterlogging than shoot parameters (shoot height and biomass). Culm height generally increased in stressed plants (by up to +24% vs. controls), while shoot biomass was significantly reduced in only two hybrids. Root biomass was reduced in all the hybrids, by an average of 30%, and significantly in 7 hybrids, while root length and area were even more severely reduced, by 30–55% vs. controls, depending on the hybrid. The earlier appearance of aerial roots seemed to be associated with greater root injuries. In leaves, the transcript of the PFP enzyme (phosphofructokinase), which is involved in glycolytic reactions, was markedly up-regulated (up to double the values) in half the waterlogged hybrids, but down-regulated in the others. The transcript of *CYP81D8* (ROS-related proteins) in waterlogged plants exhibited relevant increases or strong decreases in level, depending on the hybrid. The transcript of the *AOX1A* gene, coding for a mitochondrial respiratory electron transport chain-related protein, was markedly down-regulated in all the treated hybrids. Expression analysis of these genes under extreme waterlogging only partially correlate with the shoot and root growth impairments observed, and *AOX1A* seems to be the most informative of them.

## Introduction

Both hypoxia and anoxia are severe abiotic stresses that severely limit growth and development in many crops worldwide. Maize is very sensitive to excessive soil moisture resulting from abundant rainfall, a shallow water table or heavy soils (Zaidi et al., 2004; Lone and Warsi, 2009). In South Asia, more than 15% of total maize production is affected by floods. In India, excessive soil moisture is estimated to cause an average 25–30% loss of national maize production almost every year, while in United States waterlogging accounted for 70% of yield losses in 2011 (Zaidi et al., 2004; Lone and Warsi, 2009; Bailey-Serres et al., 2012). As climate change is expected to further exacerbate the frequency and intensity of flooding events, there is a need for greater knowledge of the plant’s mechanisms of adaptation to waterlogging.

Gas diffusivity is 10^4^-fold slower in water than in air, and oxygen dissolved in water is quickly depleted by plant root respiration and soil microorganisms resulting in hypoxic conditions. Oxygen deficiency in soils has several negative effects: it alters the nitrogen pathways, reduces nutrient availability and pH (Zaidi et al., 2004; Abiko et al., 2012; Bailey-Serres et al., 2012), and increases the solubility of toxic metals (Setter et al., 2008; Herzog et al., 2016). Respiration is the plant physiological process most sensitive to flooding. Molecular oxygen is a terminal electron acceptor in the mitochondrial electron transport chain (ETC) and in the oxidative phosphorylation process; it enables plants to generate sufficient chemical energy stored as adenosine triphosphate (ATP), which is needed for intracellular physiological and biochemical reactions. An effect of both hypoxia and anoxia is a lack of the electron acceptors that promote anaerobic respiration patterns through the activity of alcohol dehydrogenase (ADHase), the most widely studied enzyme involved in fermentation processes (Liao and Lin, 2001; Ren et al., 2014). This is the process by which flooding impairs plant growth, reduces yields and can even cause plant death.

Plant responses to flooding vary according to the duration of root submergence, soil and air temperature, plant growth stage and specific genotype tolerance. Several studies have observed that plant growth impairments and grain yield losses are greatest when flooding occurs at early growth stages (Kanwar et al., 1988; Ren et al., 2014; McDaniel et al., 2016; Yamauchi et al., 2018). However, cereals have developed morpho-physiological adaptations in response to flooding, like increased amylolytic activities in rice seedling to sustain coleoptile elongation, as well as increased production of α-amylase in maize caryopses to avoid sugar starvation, and formation of aerenchyma in maize and barley roots (Guglielminetti et al., 2000; Pompeiano et al., 2013; Yamauchi et al., 2018). A better understanding of the changes in plant morphology that take place when extreme waterlogging events occur will help identify useful morphological markers for screening tolerant genotypes. Morphological responses are driven by adjustments of gene expressions responsible for adaptation to low-oxygen regimes. The molecular mechanisms of flooding tolerance have been more extensively investigated in tolerant species, like *Oryza sativa* L., with the ethylene-response-factor-like genes SUBMERGE1 (*Sub1*) (Xu et al., 2006), SNORKEL (*SK*) (Hattori et al., 2009), and qAG-9-2 (Angaji et al., 2010; Kretzschmar et al., 2015). Only in recent years has molecular characterization of the tolerance to flooding mechanism been more widely extended to other relevant species, like *Hordeum vulgare* L. (Mendiondo et al., 2016), *Brachypodium distachyon* L. Beauv. (Rivera-Contreras et al., 2016), and *Zea mays* L. (Campbell et al., 2015). A flooding tolerance QTL named Submerge Tolerance 6 (*Subtol6*) has been recently mapped to chromosome 6 of maize (Campbell et al., 2015). *Subtol6* seems to include six genes involved in abiotic stress responses, hypoxia and senescence/oxidative stress. Two of them, RELATED TO ABA-INSENSITIVE3 (*ABI3*)/VIVIPARUS1 (*RAV1*) and HEMOGLOBIN2 (*HB2*) show differential expressions between sensitive and tolerant maize lines, suggesting their possible role as marker genes for tolerance. Besides Subtol6, other genes show differential expression after short-term submergence stress (Campbell et al., 2015): ALTERNATIVE OXIDASE 1A (*AOX1A*; Zm00001d002436), *WRKY6* maize ortholog (Zm00001d039245), *CYP81D8* (Zm00001d012322), a putative PYROPHOSPHATE-DEPENDENT FRUCTOSE-6-PHOSPHATE 1-PHOSPHOTRANSFERASE (*PFP*; JQ522972.1), PYRUVATE DECARBOXYLASE3 gene (*PDC3*; Zm00001d028759) and a gene encoding ALCOHOL DEHYDROGENASE1 (*ADH1*; Zm00001d033931).

Three out of the genes identified in the study of Campbell et al. (2015), *AOX1A*, *CYP818D8*, and *PFP*, are related to respiration and energy-production processes, that are compromised under anoxia conditions and are expected to be informative of plant tolerance to waterlogging as well. *CYP81D8* is a gene codifying for cytochrome P450, whose expression profile various studies have found to be stress-related, while its involvement in waterlogging stress tolerance has been reported by a few authors (Xu et al., 2001; Glombitza et al., 2004; Narusaka et al., 2004; Campbell et al., 2015). The genes *PFP* and *AOX1A* have received greater attention than *CYP81D8* only in recent years, and various studies (Campbell et al., 2015; Dwivedi, 2015; Gupta et al., 2015) have ascertained their involvement in flooding tolerance. The PFP enzyme can operate alternatively as a non-ATP-requiring enzyme and an ATP-dependent phosphofructokinase to catalyze the interconversion between fructose-6-phosphate and fructose-1,6-biphosphate in glycolysis reactions. *AOX1A*, the only form of alternative oxidase in monocot species (Considine et al., 2002), contributes to the maintenance of the ETC and the tricarboxylic acid cycle (TCA), pathways that are slowed down as a consequence of the increased NADH/NAD^+^ and ATP/ADP ratios (Gupta et al., 2015). *AOX1A* is also known to prevent the over-reduction in respiratory chain components that might occur after the production of harmful reactive oxygen species (ROS), thus playing an important role in avoiding cell damage by ROS (Dwivedi, 2015).

In this study, 19 commercial maize hybrids and the inbred line B73 were cultivated under extreme waterlogging conditions (6 continuous days) during early growth stages and compared with untreated controls. The aim of this study was (i) to measure the effects on shoot and root growth in order to assess the extent of tolerance to extreme waterlogging conditions in this large set of hybrids; (ii) to assess the expression analysis of the three candidate marker genes for anoxia tolerance *AOX1A*, *CYP818D8* and *PFP*, and verify if they are informative also for the hypoxic conditions of extreme waterlogging; and (iii) to identify useful morphological markers in screening tolerant genotypes. Compared with the experiment of Campbell et al. (2015) on maize submergence, in this study waterlogging was also functional to avoid interactions with other stressors (e.g., plant/water overheating), which may mask gene expression and morphological responses.

## Materials and Methods

### Experimental Set-Up

The experiment was carried out in June 2016 at the “Lucio Toniolo” experimental farm of the University of Padua, Italy (45°21′ N, 11°58′ E, 6 m a.s.l.). Seeds of 19 commercial maize (*Z. mays* L.; Supplementary Table S1) hybrids and the inbred line B73, used as reference for evaluating the efficiency of primer amplification, were sown in 4-L black PVC pots (18 cm high, 17 cm superior diameter) (4 seeds/pot) filled with 4 kg of a 1:1 (w/w) mixture of silty loam soil and sand, and fertilized with an N-P-K granular fertilizer at a rate which mimicked pre-sowing fertilization of maize (150 kg ha^-1^ K_2_O, 75 kg ha^-1^ P_2_O_5_, and 50 kg ha^-1^ N). Sowing occurred on 28 June and complete germination and emergence were recorded within 5–6 days.

Following a randomized experimental design, pots were placed in a greenhouse with 16/8 h and 24/18°C day/night conditions, and 70% RH. They were irrigated with 300 mL of water every 2 days until 11 days after sowing (DAS). In order to apply waterlogging, the pots were transferred to a tank filled with water to impose flooding for 6 days, from 11 DAS (stage BBCH 13) to 17 DAS (BBCH 15), and flooded pots were cut on the top edge (2–3 cm^2^) to allow a thin layer of water of only ∼5 mm to remain above the soil surface. To prevent water overheating during the daytime, the pots were protected with a shading net which kept the water temperature below 20°C. The experiment consisted of three pots/replicates per genotype/treatment (120 pots in total).

### Morphological Parameters

Shoot morphological parameters were recorded on three plants per pot, and three pots per hybrid-treatment (*n* = 3). At the end of the experiment (17 DAS), chlorophyll content was estimated on the last fully expanded leaf, i.e., the 5th, using a SPAD-502 chlorophyll meter (Konica-Minolta, Hong Kong). Two measures were taken from each plant, one at 1/3 and one at 2/3 the leaf length, then averaged with those from the other plants in the same pot to obtain one value per replicate.

At the end of the experiment (17 DAS), the maize plants were collected and the shoots separated from the roots. Shoot height was obtained by analyzing digital images at 300 DPI resolution with the Gimp 2.8 software, according to the leaf collar method (Abendroth et al., 2011). Shoot dry weight was recorded after oven drying at 105°C for 24 h.

Roots from the three plants in each pot were washed and separated from soil particles with a hydraulic centrifugation device and collected in a 500-μm mesh sieve. Morphological parameters were recorded through image analysis. Roots were digitized with an EPSON Expression 11000KL PRO scanner (Epson, Suwa, Japan) in binary format (1-bit) at 400 DPI resolution. Root images were then analyzed with the KS 300 ver. 3.0 software (Carl Zeiss Vision GmbH, Munich, Germany) to obtain root length, area and diameter, according to Vamerali et al.’s (2003) method.

### Leaf Sampling and cDNA Synthesis

Following phenotypic characterization of the 19 hybrids, 10 with contrasting responses to flooding stress (on a scale of shoot and root injury severity) were selected for gene expression analysis: P1733, P1570, P1547, LOLITA, P1535, P1028, P1134, SY HYDRO, DKC6752, and DKC6664, together with the inbred line B73.

Gene expression analysis was performed on leaf tissues, as non-destructive and timeliness procedure compared to root sampling. This also allowed to relate expression analysis to morphological traits of intact shoots.

RNA extraction was performed at the end of the 6-day period of waterlogging, with the aim of identifying molecular markers with stable expression over a prolonged/extreme hypoxic condition. Two-cm^2^ tissue samples from the third leaf of the three plants of each replicate were collected, immediately frozen in liquid nitrogen and stored at -80°C until further processing. Total leaf RNA was extracted from submerged and control plants with the TRIzol^®^ Reagent (Thermo Fisher Scientific), according to the manufacturer’s protocol. RNA concentration was verified with NANODROP 2000c (Thermo Fisher Scientific). One microgram of total RNA from each sample was reverse-transcribed to cDNA in a 20 μL reaction volume using SuperScript III^TM^ Reverse Transcriptase (Thermo Fisher Scientific), according to the manufacturer’s instructions.

### Real-Time Quantitative PCR (qRT-PCR) Analysis

Expression analysis was performed on three genes, among others from Campbell et al.’s (2015) studies, having the highest efficiency of primer amplification (without problems linked to the sequence diversity) according to a preliminary test: *AOX1A* (Zm00001d002436), *CYP81D8* (Zm00001d012322), and a putative *PFP* (JQ522972.1). The three marker genes were tested by qRT-PCR. Specific primers were selected from Campbell et al. (2015, Supplementary Table S2) and first evaluated in the inbred line B73 to assess their amplification efficiency.

For each gene, 3 biological replicates (each derived from the 3 plants of each pot) and 2 technical replicates were analyzed using 4 μL of cDNA samples diluted 1:5 (*AOX1A* and *CYP81D8*) or 1:20 (*PFP*) in 20 μL reaction mixture containing 2× Power SYBR^TM^ Green PCR Master Mix (Applied Biosystems). The analyses were performed with the StepOne^TM^ and StepOnePlus^TM^ Systems (Applied Biosystems). Real-time conditions were: 20 s at 95°C, 40 cycles of 3 s at 95°C, and 30 s at 60°C. For each reaction, the product melting curve was generated by heating from 60 to 95°C in increments of 0.2/s°C. The constitutively expressed *EF1-α* gene was used as the housekeeping internal control of the cDNA quantity. Relative quantification of gene expressions [normalized to EF1-α transcript quantities, selected from Lin et al. (2014, Supplementary Table S2)] was performed with the Applied Biosystems 7500 ver. 2.0.5 software and the ΔΔCT method.

### Statistical Analysis

The data from the morphological parameters examined in waterlogged plants and untreated controls were subjected to an ANOVA using the Statgraphics Centurion XVII software (Adalta, Arezzo, Italy). Separation of means was set at *P* ≤ 0.05 with the Newman–Keuls test. Significant differences between treatments are indicated with asterisks in the figures below.

Factorial discriminant analysis [Multigroup Discriminant Analysis (MDA) with Wilks’ lambda and Pillai’s trace tests], and principal component analysis (PCA) were also carried out. MDA allowed us to describe the changes in morphological traits in response to hypoxia, and PCA to describe the relationship between the morphological changes and the expressions of the three genes examined. Before analysis, multivariate data normality was verified by the Shapiro test, and data were standardized by subtracting the mean and dividing the result by the standard deviation for each variable. All analyses were performed in MS Excel XLSTAT (Addinsoft, Paris, France).

## Results

### Shoot Growth Parameters

#### SPAD Readings

**FIGURE 1:**
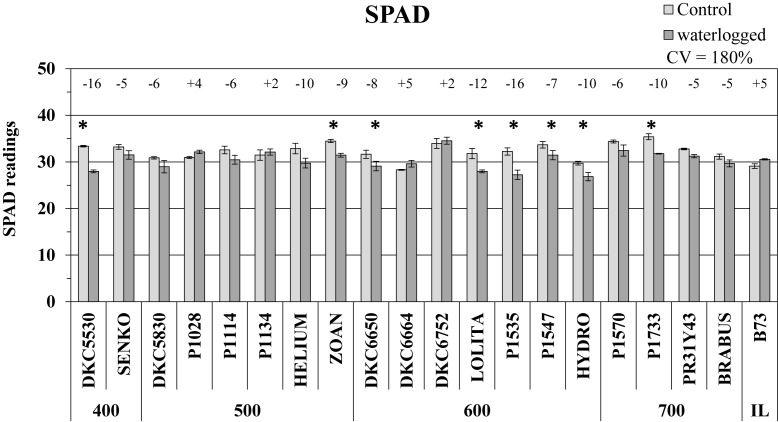
SPAD readings (average SPAD units ± SE; *n* = 3) from control and waterlogged plants of commercial hybrids and the inbred line B73 at 17 days after sowing (DAS), grouped according to FAO class of maturity (from 400 to 700). Numbers above histograms indicate the percentage variation between waterlogged plants and controls for each hybrid, with asterisks indicating significance (*P* ≤ 0.05).

SPAD values, which represent the leaf chlorophyll content, were generally lower in the plants subjected to 6 days of waterlogging than in untreated controls, with almost 50% of the hybrids showing a significant decrease (Figure 1). Considerable phenotypic variability in response to waterlogging was observed among hybrids (CV = 180%), with the largest decreases in SPAD recorded in the DKC5530 and P1535 hybrids (both -16% vs. respective controls), while some others, i.e., P1028, P1134, DKC6664, and DKC6752, as well as the inbred line B73 exhibited only slight increases, up to a maximum of 5%.

#### Culm Height

**FIGURE 2:**
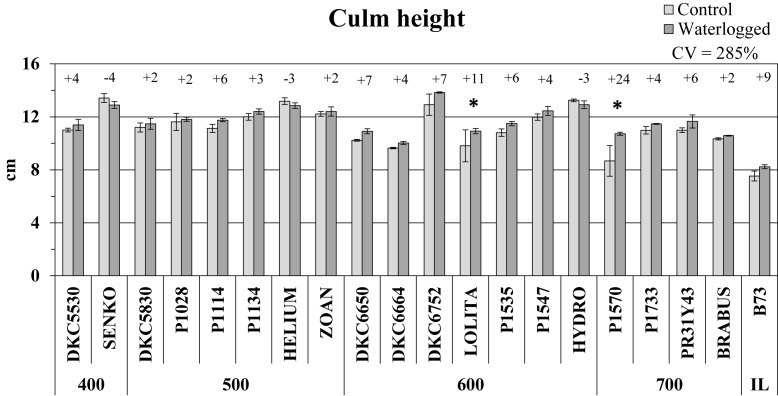
Culm height (average ± SE; *n* = 3) of control and waterlogged plants of commercial hybrids and the inbred line B73 at 17 DAS, grouped according to FAO class of maturity (from 400 to 700). Numbers above histograms indicate the percentage variation between waterlogged plants and controls for each hybrid, with asterisks indicating significance (*P* ≤ 0.05).

Culm height generally increased in plants affected by waterlogging compared to controls (Figure 2), but the increase was significant (*P* ≤ 0.05) in only two hybrids, P1570 (+24%) and LOLITA (+11%). The inbred line B73 also exhibited a 9% increase in culm height under waterlogging, whereas SY SENKO, SY HELIUM, and SY HYDRO exhibited a decrease, albeit slight (-4, -3, and -3%, respectively, vs. respective controls).

#### Shoot Biomass

**FIGURE 3:**
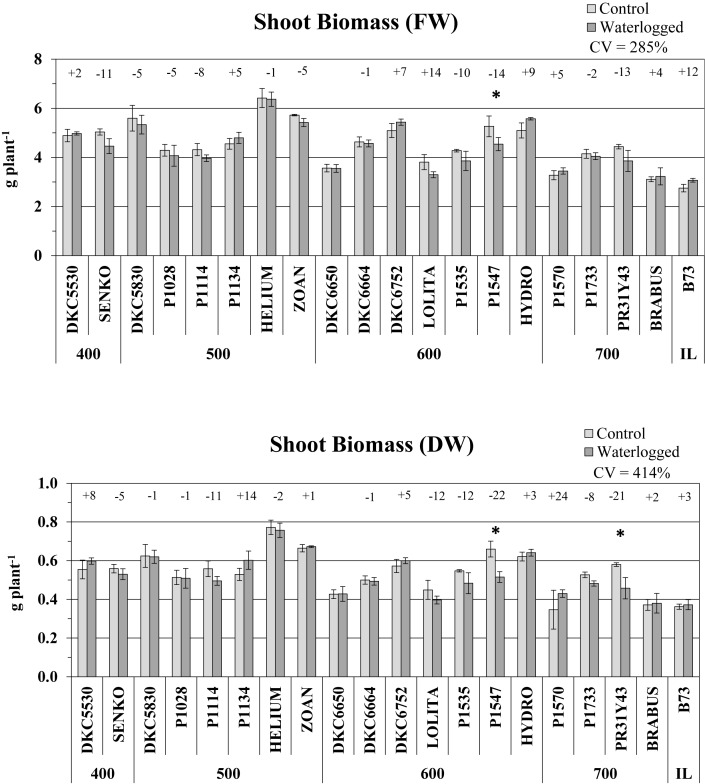
Shoot fresh weight (FW) and dry weight (DW) (average ± SE; *n* = 3) of control and waterlogged plants of commercial hybrids and the inbred line B73 at 17 DAS, grouped according to FAO class of maturity (from 400 to 700). Numbers above histograms indicate the percentage variation between waterlogged plants and controls for each hybrid, with asterisks indicating significance (*P* ≤ 0.05).

Shoot biomass was weakly affected by the waterlogging stress imposed. A slight decrease in shoot fresh weight was observed in 12 hybrids, but was significant only for P1547 (-14% vs. controls, *P* ≤ 0.05) (Figure 3). Shoot dry weight followed the same trend as fresh weight, their variations in control vs. waterlogged conditions correlating positively (*R*^2^ = 0.60; *P* ≤ 0.05) and involving the same hybrids. Waterlogging significantly decreased the shoot DW of only two hybrids, P1547 and PR31Y43 (-22 and -21%, respectively, vs. controls, *P* ≤ 0.05) (Figure 3). There were increases in shoot DW in the other hybrids, with the maximum variation (+24%) found in P1570 (like culm height), but none was significant. The variations in shoot DW in response to waterlogging were higher than in FW and culm height (CV = 414% vs. 28 and 120%, respectively).

### Root Growth Parameters

**FIGURE 4:**
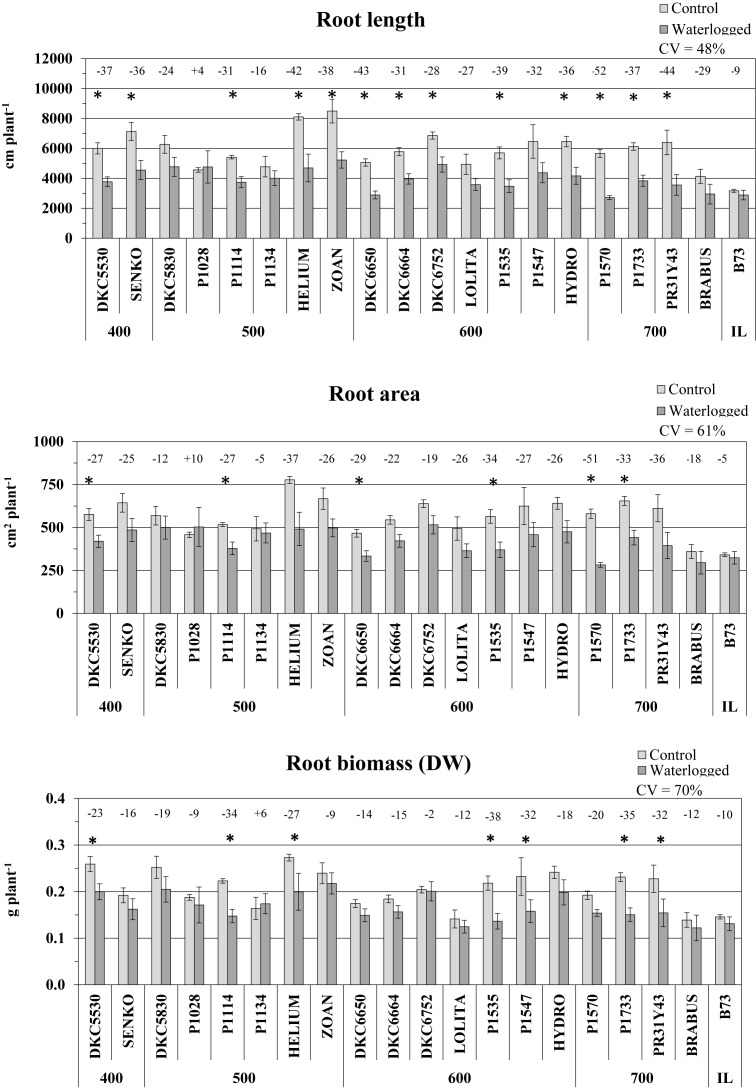
Root length (average ± SE; *n* = 3), area (average ± SE; *n* = 3), and biomass (average ± SE; *n* = 3) of control and waterlogged plants of commercial hybrids and the inbred line B73 at 17 DAS, grouped according to FAO class of maturity (from 400 to 700). Numbers above histograms indicate the percentage variation between waterlogged plants and controls for each hybrid, with asterisks indicating significance (*P* ≤ 0.05).

Root growth was more impaired by waterlogging than shoot growth. Length was the root parameter most affected, and was reduced in all the hybrids except P1028, which exhibited slightly higher values in waterlogged than in control plants (Figure 4). The reduction in root length across the whole set of hybrids was on average 31%, and was significant in 13 of them. The greatest effect was observed in P1570, which was also greatly affected above ground, followed by PR31Y43 and DK6650 (-53, -44, -43% vs. controls, respectively). Less damage was observed in P1134 and DKC5830 (-16 and -24%, respectively).

Similarly, root area was also greatly reduced by waterlogging, although to a lesser extent than root length, the decrease being 24% on average, and significant in 7 hybrids (*P* ≤ 0.05). Only one hybrid, P1028, slightly increased in root area as a consequence of hypoxia stress (Figure 4), as it did in root length. P1570, SY HELIUM, and PR31Y43 suffered the most severe decreases (-51, -37, and -36%, respectively), while P1134 and DKC5830 again exhibited the smallest reductions, as they did for root length.

Regarding root biomass (DW), this was again reduced by waterlogging in all hybrids, by an average of 18%, and significantly in 7 of them (*P* ≤ 0.05) (Figure 4). The greatest decreases were recorded in hybrids P1535, P1733, and P1114 (-38, -35, and -34%, respectively), while DKC6752, P1028, and SY ZOAN were only slightly impaired. P1134 was the only hybrid that increased its root biomass under waterlogging, although not by much (+6% vs. control).

Root diameter exhibited an opposite trend to the other root parameters under hypoxic conditions in that it generally increased, by an average of 12%, and by maximum of 21% in SY ZOAN and 25% in DKC6650, while P1570 and LOLITA remained very stable (+1% and +2%, respectively) (data not shown).

As a consequence of the plants’ responses to waterlogging, the root-to-shoot ratio was always reduced, by between 6% (hybrid P1134) and 29% (P1733 and DKC 5530), with a 58% variability (data not shown).

Of the various root parameters, root length varied the least across hybrids (CV = 48%), followed by root area and biomass (CV = 61 and 70%, respectively).

Adventitious aerial roots were observed to start growing on the 4th day of waterlogging treatment (15 DAS). On that day, aerial roots were visible in all waterlogged replicates of only one hybrid, i.e., DKC5530, in 2 of the 3 replicates of SY HELIUM and SY ZOAN, and in only 1 of the 3 replicates of SY BRABUS, DKC5830, DKC6664, PR31Y43, and P1547. Aerial roots were visible in a few other hybrids at 16 DAS (5th day of waterlogging), and in the whole set of hybrids at 17 DAS (6th day of waterlogging). The inbred line B73 was recorded as having aerial roots in 2 of the 3 replicates at 16 DAS and still only 2 at 17 DAS.

The hybrids that formed aerial roots earlier (4th day of waterlogging stress) were among the highest impaired in terms of root length, area, and biomass.

### PCA and MDA on Morphological Parameters

**FIGURE 5:**
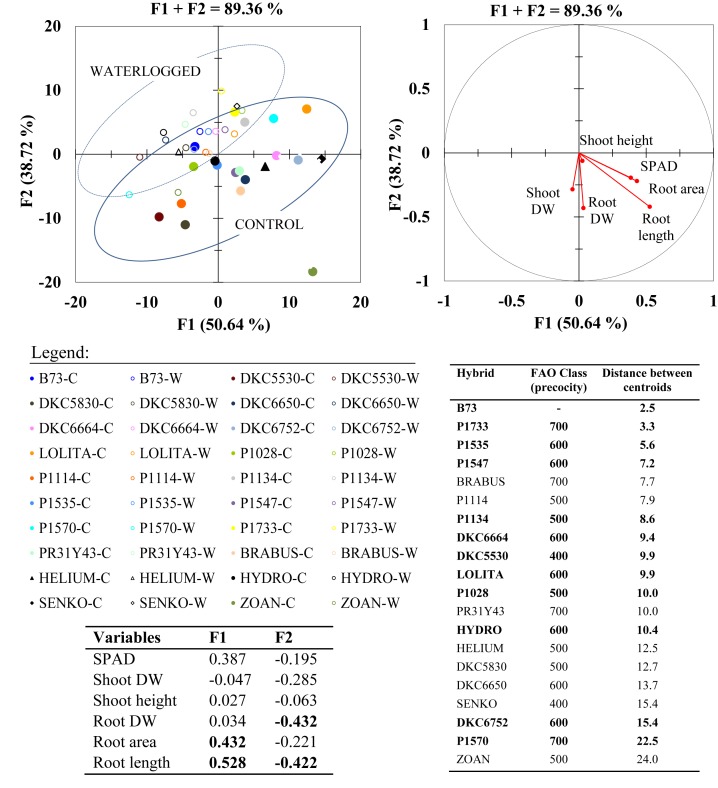
Principal component analysis (PCA; top right) for morphological shoot and root parameters with highly informative variables (loadings > | 0.4| ) in bold within synthetic variables F1 and F2 (bottom left Table); and multigroup discriminant analysis (MDA; top left) of waterlogged (W) and control (C) plants of commercial hybrids and the inbred line B73 at 17 DAS. Distances between centroids for each hybrid (waterlogged vs. control) in MDA (bottom right Table) are reported in ascending order, with highlighted hybrids (bold) considered for the subsequent molecular analysis.

Principal component analysis conducted on the data for shoot and root morphological parameters allowed us to identify two synthetic variables, which explained an overall variability of 89.36% (Figure 5). The most informative variables (loadings > |0.4|) were root length, followed by root area and biomass, suggesting that the root system is more involved in adaption to a waterlogged environment than the aboveground compartment. According to the vector direction of each variable, good correlations among variables are indicated by vectors plotted very close together in the same quadrant, as occurs between root length and SPAD, and between root length and root area.

Centroid position and cluster separation in the DA summarize the phenotypic variability in the response of maize hybrids to waterlogging stress, and show that under hypoxic conditions all the parameters studied generally decreased, but to varying extents across hybrids.

The overall variability between waterlogged and control conditions in each hybrid is summarized as the distance between the two centroids (waterlogged and control) for each hybrid (Figure 5). The decreasing values of this centroid inter-distance across the hybrids show that the morphological changes from the control to the waterlogged environment ranged from large in hybrids DKC6752 and P1570, to intermediate in SY HYDRO, P1028, LOLITA, DKC6664, and P1134, and few in the inbred line B73, followed by hybrids P1733, P1535, P1547. These 10 hybrids and the inbred line B73 were considered in the following step of this research, which was to identify gene expressions in response to waterlogging within a wide range of phenotypic variability.

### Gene Expression Analysis

**FIGURE 6:**
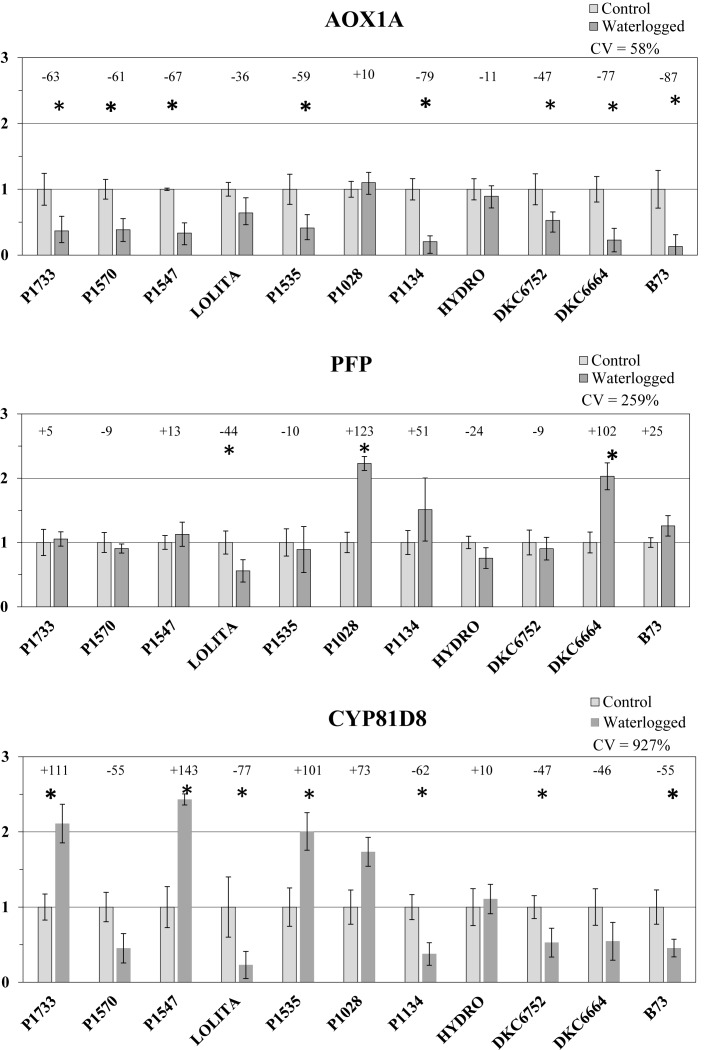
Standardized (on controls) transcript levels of genes CYP81D8, PFP, and AOX1A in waterlogged conditions (average ± SE; *n* = 3) in 10 representative hybrids and the inbred line B73. Numbers above histograms indicate the percentage variation between waterlogged plants and controls for each hybrid, with asterisks indicating significance (*P* ≤ 0.05).

In light of the results of the DA of the morphological parameters, gene expression focused on 10 maize hybrids as representative of a wide variability in phenotypic response to waterlogging treatment (Figure 6).

In leaves, after 6 days of submergence treatment, transcript qPCR analysis of *AOX1A* revealed marked down-regulation, from -10 to -80%, in all the flooded hybrids compared with untreated controls, except for the P1028 hybrid in which a slight increase occurred (+10%). There was also very large (and significant) down-regulation of the *AOX1A* gene transcript in the reference inbred line B73 (-87%). The variability among hybrids for changes in transcript expression (control vs. waterlogged conditions) was relatively high (CV = 58%).

Expression of the *PFP* transcript was up-regulated in half the waterlogged genotypes compared with respective controls, from +5% in P1733 to values >100%, with the greatest increases in those hybrids that were less impaired in the root, i.e., P1028, DK6664, and P1134. *PFP* expression was down-regulated in the other hybrids, but with smaller decreases compared with *AOX1A* gene expression (from -9% in P1570 and DKC6752 to -44% in LOLITA). The variability among hybrids in the changes in transcript expression (control vs. waterlogged conditions) was high (CV = 259%).

Lastly, the *CYP81D8* transcript of waterlogged plants showed relevant increases or strong decreases in level according to the hybrid considered. In P1547, P1733, P1535, and P1028, expression doubled under waterlogging stress (significantly except for P1028), in SY HYDRO it only slightly increased, and in all other hybrids a marked down-regulation was observed (from -46 to -77% compared with untreated controls). The variability among hybrids in changes in transcript expression (control vs. waterlogged conditions) was also very high in this case (CV = 927%).

### PCA and MDA of Morphological and Molecular Responses

**FIGURE 7:**
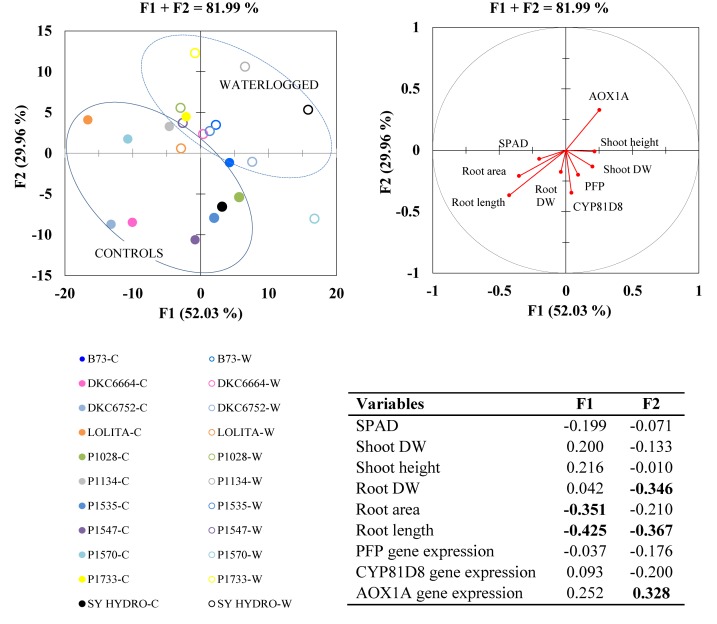
Principal component analysis (PCA; top right) with highly informative variables (loadings > | 0.3| ) in bold within synthetic variables F1 and F2 (bottom right Table); and multigroup discriminant analysis (MDA; top left) in waterlogged (W) and control (C) plants of 10 representative hybrids and the inbred line B73 at 17 DAS.

Principal component analysis carried out on the whole dataset, including morphological traits and gene expression data, identified two synthetic variables, which explained a large part of the overall variability (F1 + F2 = 81.99%) (Figure 7). Root parameters again explained more variability than shoot parameters, root length being the most representative (loadings: F1 = 0.43; F2 = 0.37). Of the three genes studied, *AOX1A* was the most relevant (loadings: F1 = 0.25; F2 = 0.33). *PFP* and *CYP81D8* seemed to be positively correlated with root and shoot growth, as they are plotted closer together in the quadrants according to their vector direction, but they had very low loadings.

Correlation analysis of all the parameters, calculated as the differences between control and waterlogged plants for the 10 representative hybrids, revealed generally high positive and significant correlations among the variations in shoot (biomass) and root (biomass, area and length) caused by waterlogging (Table 1). However, morphological changes were poorly correlated with gene regulation. Root length correlated with up-regulation of *AOX1A* (*R*^2^ = 2.4%; *P* > 0.05) and *CYP81D1* (*R*^2^ = 0.7%; *P* > 0.05), and with down-regulation of *PFP* (*R*^2^ = 14%, *P* ≤ 0.05), although none explained much variability. The down-regulation of PFP was also significantly correlated with root area (*R*^2^ = 16.6%) and SPAD (*R*^2^ = 21.6%), while the up-regulation of *CYP81D1* significantly correlated with shoot and root biomass (*R*^2^ = 19.9 and 24.5%, respectively). In general, up-regulation of *CYP81D8* correlated better with root biomass, whereas down-regulation of *PFP* correlated better with root area and length (*P* ≤ 0.05; Table 1).

**Table 1:** Correlation coefficients (r) among the differences (control – waterlogged) for morphological and gene expression variables, with significant correlations in bold (*P* ≤ 0.05).

Variables	SPAD	Shoot biomass	Shoot height	Root biomass	Root area	Root length	PFP gene	CYP81D8 gene	AOX1A gene
SPAD	–	0.323	0.029	**0.543**	**0.532**	**0.475**	**–0.465**	0.288	0.138
Shoot biomass		–	0.165	**0.583**	0.269	0.267	–0.037	**0.446**	0.021
Shoot height			–	0.267	0.039	0.110	0.163	0.233	0.278
Root biomass				–	**0.768**	**0.746**	–0.164	**0.495**	0.172
Root area					–	**0.979**	**–0.407**	0.069	0.137
Root length						–	**–0.376**	0.084	0.155
PFP gene							–	0.305	–0.195
CYP81D8 gene								–	0.154
AOX1A gene									–


## Discussion

Waterlogging is a severe abiotic stressor causing significant growth impairment and yield losses in maize, with different intensities according to the severity and duration of the anoxic/hypoxic conditions, the phenological stage of the plant and the sensitivity of the genotype. In the present study, an extreme flooding condition imposed on maize plants at early growth stage severely impaired all the shoot and root morphological parameters examined, although root growth was more impaired than shoot growth. Root length, area and biomass were significantly reduced after 6 days of waterlogging in almost all the hybrids, with one exception, but to different extents according to genotype-specific sensitivity. The reduction in length ranged from -16% to as much as -52% compared with controls over this short stress period. Aboveground plant parameters were less sensitive to waterlogging, but variability was very high due to either positive or negative variations in shoot biomass associated with a general increase in plant height, probably because oxygen was better diffused in the shoot tissues of some hybrids than in others (Armstrong et al., 1994). As a consequence, leaf chlorophyll content was also generally compromised in the waterlogging treatment (on average -6%) as a consequence of reduced synthesis and increased oxidation of pigments (Yan et al., 1996; Zheng et al., 2009). According to the effect on shoot and root growth, the inbred line B73 showed appreciable tolerance to waterlogging, similarly to the studies of Mano et al. (2002, 2006).

Early vegetative stages are the most susceptible to excessive soil moisture, whereas well-developed maize plants suffer less damaged from similar stress conditions (Kanwar et al., 1988; Zaidi et al., 2004). McDaniel et al. (2016) observed that during the V4 stage, a 3-day period of flooding led to the immature nodal roots and most of the small, less active primary roots dying – an effect that would explain the substantial root injuries to our plants after 6 days of flooding – whereas non-significant root mortality was recorded between the V12 and R1 stages. Flooding stress imposed at early vegetative growth is known to translate into severe reductions in plant height, dry matter production and yield at maturity, with plants exhibiting a dwarfing effect, which varies according to the duration of the waterlogging (Zaidi et al., 2004; Ren et al., 2014). As in our study shoot height did not decrease, but even increased in a couple of hybrids, we suspect this is a transient effect, which will reverse in later stages as a result of permanent plant damage. Indeed, culm elongation has been reported to be a common strategy for withstanding stress conditions (Bailey-Serres et al., 2012). According to these authors, when hypoxia is imposed on maize roots, ethylene concentration increases in root tissues, thereby altering the phytohormonal balance and leading to the onset of the “low oxygen escape strategy,” which consists in extending the culm to compensate for the alterations caused by flooding.

As oxygen is rapidly depleted in water of saturated soil (within 24–36 h), the prolonged period of flooding in this study caused substantial root damage and probably reduced the variability among hybrids compared with shoot response, in agreement with the results of Mano et al. (2005), Mano and Omori (2007), and Abiko et al. (2012). The general increase in root diameter (up to +25%) observed after 6 days of waterlogging might also be a morphological change linked to the formation of aerenchyma, commonly reported in maize and barley (Rajhi et al., 2011; Herzog et al., 2016; Yamauchi et al., 2018), as a key adaptation to flooding. Although less efficient than schizogenous aerenchyma in rice, the formation of lysigenous aerenchyma, with its poorly specialized intercellular spaces, enables the internal movement of gasses in plant roots, petioles and stems. This aerenchyma has been found to start forming between 18 and 24 h after waterlogging treatment, and to be ethylene mediated (Rajhi et al., 2011; Bailey-Serres et al., 2012; Yamauchi et al., 2018).

Similarly, adventitious root formation is reported to be an adaptive strategy to compensate for growth inhibition or even death of distal portions of roots during waterlogging (Sauter, 2013; Yamauchi et al., 2018). Having applied waterlogging stress for an uninterrupted 10-day period at various maize growth stages, Zaidi et al. (2004) found the number of newly developed adventitious roots to be the most notable morphological change they observed: the number of adventitious roots increased in all the genotypes investigated, but to a greater extent in the more tolerant ones. They also observed that early increased adventitious rooting during waterlogging was closely related to final grain yield, allowing them to conclude that this morphological trait can be profitably used as a selection criterion for flooding tolerance in maize. In our study, the earlier appearance of aerial roots seemed to be associated with greater plant injuries, although we did not count the number of aerial roots, nor were the plants grown to maturity, so we cannot directly compare our results with those of Zaidi et al. (2004).

As an overall evaluation of the morphological responses of hybrids, it was not possible to establish a relationship between growth impairment under waterlogging and the FAO class of maturity, although growth was generally slightly earlier/higher in non-waterlogged plants of early-season hybrids compared with late-season hybrids.

The set of hybrids investigated in this study was sufficiently large to evidence considerable variability in their morphological responses to waterlogging, and suitable for relating to gene expression. Some useful indications to better identify waterlogging-tolerant hybrids were obtained. However, molecular characterization for mechanisms of tolerance to anoxia/hypoxia has only recently been broadened to include maize (Voesenek and Bailey-Serres, 2015), but investigations have concerned only inbred reference lines. On the basis of a transcriptome analysis in roots of a tolerant inbred line, Arora et al. (2017) has recently demonstrated existing 21,364 differentially expressed genes (DEGs) under waterlogging stress conditions, which regulate relevant pathways for energy-production, programmed cell-death (PDC), aerenchyma formation and ethylene responsiveness. In this study we aimed at identifying useful morphological and molecular markers for screening rapidly large sets of genotypes. In this view, we investigated whether there was a correlation between the morphological changes observed and expression of the three putative marker genes reported in recent studies (Campbell et al., 2015). In the research of Campbell et al. (2015), as in other recent studies (Guo et al., 2011; Gupta et al., 2015), *CYP81D8*, *PFP*, and *AOX1A* are proposed as marker genes to identify submergence tolerance lines.

Under submergence, *CYP81D8*, a stress-related gene codifying for cytochrome P450, was significantly down-regulated in tolerant lines compared with sensitive ones (Xu et al., 2001; Glombitza et al., 2004; Narusaka et al., 2004; Campbell et al., 2015). The expression of *PFP*, involved in glycolytic reactions (Dwivedi, 2015), was strongly reduced by submergence in all the maize lines studied by Campbell et al. (2015), although in our study *PFP* transcript abundance was higher in the hybrids more tolerant to extreme waterlogging. *AOX1A*, known for its contribution to the maintenance of the ETC and the tricarboxylic acid cycle (TCA), was down-regulated in maize hybrids with higher tolerance to submergence (Dwivedi, 2015; Gupta et al., 2015).

In our study, waterlogged plants showed contrasting variations in *CYP81D8* transcript levels, both relevant increases and strong decreases, depending on the hybrid considered. The expression level of the PFP enzyme was also up-regulated in half the waterlogged hybrids and down-regulated in the others. In contrast, qPCR analysis of *AOX1A* revealed important down-regulation of this transcript in all waterlogged hybrids, except P1028, compared to controls (from -10 to -80%). The expression of this gene was also significantly down-regulated in the inbred line B73, suggesting that this trait can be incorporated into commercial hybrids. Discriminant analysis confirmed *AOX1A* as the most informative of the three genes investigated under waterlogging conditions.

Morphological parameters (damage to shoot and root growth) did not correlate highly with the expression levels of the putative marker genes *CYP81D8*, *PFP*, and *AOX1A*, and it was not possible to discriminate clearly between the more tolerant and susceptible hybrids. We may argue that these genes, codifying for proteins related to physiological processes, like the respiratory chain, glycolytic reaction, and ROS production, do not completely explain the morphological changes observed. As these genes were instead well correlated with the changes in plant morphology observed by Campbell et al. (2015) under submergence, we may conclude that they are less informative under waterlogging. However, the good level of tolerance of some hybrids, like P1028, which was associated with significant up-regulation of PFP, also suggests that there probably is large genetic variability among hybrids for this trait, which hampered identification of a general rule/relationship between phenotypic response and gene transcript level.

## Conclusion

When a period of extreme flooding is imposed at the early growth stage of maize, greater growth impairment is detectable in the root than in the shoot. As we found large variability in the responses of shoot traits, and of root traits, although to a lesser extent, in the set of commercial hybrids we studied, there is reasonable scope for screening for waterlogging stress tolerance, although the growth and yield of mature plants should be verified in further studies. The expressions of *CYP81D8*, *PFP*, and *AOX1A* genes, codifying for proteins related to essential physiological processes, explained only partially the shoot and root damage observed, suggesting that genetic variability in waterlogging tolerance in current commercial hybrids is probably small, and/or other candidate genes should be investigated under hypoxic stress conditions. Meanwhile, *AOX1A*, codifying for an alternate oxidase involved in the respiratory chain, which was clearly down-regulated in almost all the hybrids under extreme waterlogging, can be used for screening currently available genotypes. Although *AOX1A* turned out to be the most informative gene in explaining morphological responses across hybrids (by discriminant analysis), up-regulation of *CYP81D1* and down-regulation of PFP should also be considered for preserving root growth, which showed the greatest impairment by waterlogging.

## Author Contributions

TV and SV conceived the research. CDC and MF recorded the morphological parameters. BV performed the qRT-PCR analysis. CDC, AP, and BV analyzed the data. AP, CDC, SV, and TV wrote the manuscript. All authors read and approved the final manuscript.

## Conflict of Interest Statement

The authors declare that the research was conducted in the absence of any commercial or financial relationships that could be construed as a potential conflict of interest.
