# Supplementary material for: Morphological Changes and Expressions of AOX1A, CYP81D8, and Putative PFP Genes in a Large Set of Commercial Maize Hybrids Under Extreme Waterlogging
Source: Front Plant Sci. 2019 Feb 4;10:62. doi: 10.3389/fpls.2019.00062 (PMC6369177; doi:10.3389/fpls.2019.00062)
Supplement: Supplementary file 1 [file Table_1.docx]

Supplementary Table S1. List of the commercial maize (*Zea mays* L.) hybrids considered along with the inbred line B73.

|  | Hybrid | Seed Company | FAO Class  (precocity) |
| --- | --- | --- | --- |
| 1 | PR31Y43 | Pioneer Hi-Bred | 700 |
| 2 | P1733 | Pioneer Hi-Bred | 700 |
| 3 | P1570 | Pioneer Hi-Bred | 700 |
| 4 | P1547 | Pioneer Hi-Bred | 600 |
| 5 | LOLITA | Pioneer Hi-Bred | 600 |
| 6 | P1535 | Pioneer Hi-Bred | 600 |
| 7 | P1028 | Pioneer Hi-Bred | 500 |
| 8 | P1134 | Pioneer Hi-Bred | 500 |
| 9 | P1114 | Pioneer Hi-Bred | 500 |
| 10 | SY SENKO | Syngenta | 400 |
| 11 | SY HELIUM | Syngenta | 500 |
| 12 | SY HYDRO | Syngenta | 600 |
| 13 | SY BRABUS | Syngenta | 700 |
| 14 | SY ZOAN | Syngenta | 500 |
| 16 | DKC5530 | Dekalb | 400 |
| 15 | DKC5830 | Dekalb | 500 |
| 17 | DKC6752 | Dekalb | 600 |
| 18 | DKC6664 | Dekalb | 600 |
| 19 | DKC6650 | Dekalb | 600 |
| 20 | B73 | - | - |
