# Supplementary material for: Morphological Changes and Expressions of AOX1A, CYP81D8, and Putative PFP Genes in a Large Set of Commercial Maize Hybrids Under Extreme Waterlogging
Source: Front Plant Sci. 2019 Feb 4;10:62. doi: 10.3389/fpls.2019.00062 (PMC6369177; doi:10.3389/fpls.2019.00062)
Supplement: Supplementary file 2 [file Table_2.docx]

Supplementary Table S2. Accession ID and forward (Fw) and reverse (Rev) primer sequences of the housekeeping internal control EF1-α and of the three marker genes considered in this study.

|  | Gene | Accession ID | Primer sequence (5’–3’) | Size (pb) | | Reference |
| --- | --- | --- | --- | --- | --- | --- |
| Housekeeping internal control | EF1-α | NM_001112117 | Fw: TGGGCCTACTGGTCTTACTACTGA | 135 | Lin et al. (2014) | |
|  |  |  | Rev: ACATACCCACGCTTCAGATCCT |  |  |  |
| Marker genes tested | AOX1A | GRMZM2G125669 | Fw: AGTCGCTGCGCTTCCCCA | 324 | Campbell et al. (2015) | |
|  |  |  | Rev: CGACGCGGTGCGCGAACT |  |  |  |
|  | CYP81D8 | GRMZM2G087875 | Fw: GTGCGGCCAACCTGTGGA | 341 |  |  |
|  |  |  | Rev: GTTAAGCAAGAGCGACATTGC |  |  |  |
|  | PFP | TCONS_00005048 | Fw: GCGATTATGGCTACGTTCTTGG | 202 |  |  |
|  |  |  | Rev: CCATGTGGACAGCAGGCTTA |  |  |  |
